# How Does Religious Value Similarity Shape Mother-Adult Child Relations Over Time in Later-Life Families

**DOI:** 10.1093/geroni/igaf122.954

**Published:** 2025-12-31

**Authors:** Ranran He, J Jill Suitor, Destiny Ogle, Robert Frase, Megan Gilligan, Di Wang

**Affiliations:** Purdue University, West Lafayette, Indiana, United States; Purdue University, West Lafayette, Indiana, United States; Purdue University, West Lafayette, Indiana, United States; Southern Illinois University, Carbondale, Illinois, United States; University of Missouri, Columbia, Missouri, United States; Purdue University, West Lafayette, Indiana, United States

## Abstract

Fueled by increasing political polarization and broader trends toward secularization, generational value discrepancies in religious beliefs expanded in the United States over the past decade. Despite these general trends, it remains unclear whether parents and adult children within the same families changed their perceptions regarding the extent to which they shared religious values. Further, it is not known whether similarity of religious values played the same role in the quality of parent-adult child relations in later-life families across this period. To address these questions, we used data collected from 291 adult children from Time 2 (2008–2011) and Time 3 (2020–2021) of the Within-Family Differences Study to examine (1) patterns of stability and changes in midlife adult children’s perceptions of religious similarity to their older mothers, and (2) changes in the effects of perceived religious similarity on mother-child closeness and tension. Our findings suggest that adult children’s perceptions of religious similarity with their mothers remained relatively stable over time in later-life families. Perceived religious value similarity with mothers had a positive effect on closeness with mothers at both waves. Although similarity in religious values had no discernible effects on mother-child tension at T2, a strong negative association was found at T3. There were no differences in the associations by child’s gender for either closeness or tension at T2, or for closeness at T3; however, at T3, religious similarity was negatively associated with tension for daughters, but not sons.

